# Paleohistology of *Susisuchus anatoceps* (Crocodylomorpha, Neosuchia): Comments on Growth Strategies and Lifestyle

**DOI:** 10.1371/journal.pone.0155297

**Published:** 2016-05-05

**Authors:** Juliana M. Sayão, Renan A. M. Bantim, Rafael C. L. P. Andrade, Flaviana J. Lima, Antônio A. F. Saraiva, Rodrigo G. Figueiredo, Alexander W. A. Kellner

**Affiliations:** 1 Laboratório de Biodiversidade do Nordeste, Centro Acadêmico de Vitória, Universidade Federal de Pernambuco.Vitória de Santo Antão, Pernambuco, Brazil; 2 Laboratório de Paleontologia, Universidade Regional do Cariri.Crato, Ceará, Brazil; 3 Departamento de Ciências Biológicas, Universidade Federal do Espírito Santo. Vitória, Espírito Santo, Brazil; 4 Laboratório de Sistemática e Tafonomia de Vertebrados Fósseis, Museu Nacional, Universidade Federal do Rio de Janeiro.Rio de Janeiro, Rio de Janeiro, Brazil; University of Zurich, SWITZERLAND

## Abstract

*Susisuchus anatoceps* is a neosuchian crocodylomorph lying outside the clade Eusuchia, and associated with the transition between basal and advanced neosuchians and the rise of early eusuchians. The specimen MPSC R1136 comprises a partially articulated postcranial skeleton and is only the third fossil assigned to this relevant taxon. Thin sections of a right rib and right ulna of this specimen have been cut for histological studies and provide the first paleohistological information of an advanced non-eusuchian neosuchian from South America. The cross-section of the ulna shows a thick cortex with 17 lines of arrested growth (LAGs), a few scattered vascular canals, and primary and secondary osteons. This bone has a free medullary cavity and a spongiosa is completely absent. Thin sections of the rib show that remodeling process was active when the animal died, with a thin cortex and a well-developed spongiosa. In the latter, few secondary osteons and 4 LAGs were identified. According to the observed data, *Susisuchus anatoceps* had a slow-growing histological microstructure pattern, which is common in crocodylomorphs. The high number of ulnar LAGs and the active remodeling process are indicative that this animal was at least a late subadult, at or past the age of sexual maturity. This contradicts previous studies that interpreted this and other *Susisuchus anatoceps* specimens as juveniles, and suggests that full-grown adults of this species were relatively small-bodied, comparable in size to modern dwarf crocodiles.

## Introduction

Crocodylomorpha is the most common group of Mesozoic tetrapods in Brazil [1, 2]. Fossils described in the past years indicate that the maximum diversity of this group took place during the Cretaceous Period (145–66 Ma), when the Notosuchia dominated the ancient Brazilian terrestrial landscapes (e.g., [3–9]), along with other less specialized crocodylomorphs (e.g. [10]). On the other hand, the fossil record of Neosuchia is relatively poor for Cretaceous deposits of this country. This latter clade is often represented by dubious and poorly-preserved material such as those of “*Goniopholis paulistanus*” and “*Hyposaurus derbianus*" [11–13]. The gigantic *Sarcosuchus hartii* is an important neosuchian from Brazil; however, a revision of the material and taxonomic status of this taxon is badly needed [14, 15].

**Fig 1:**
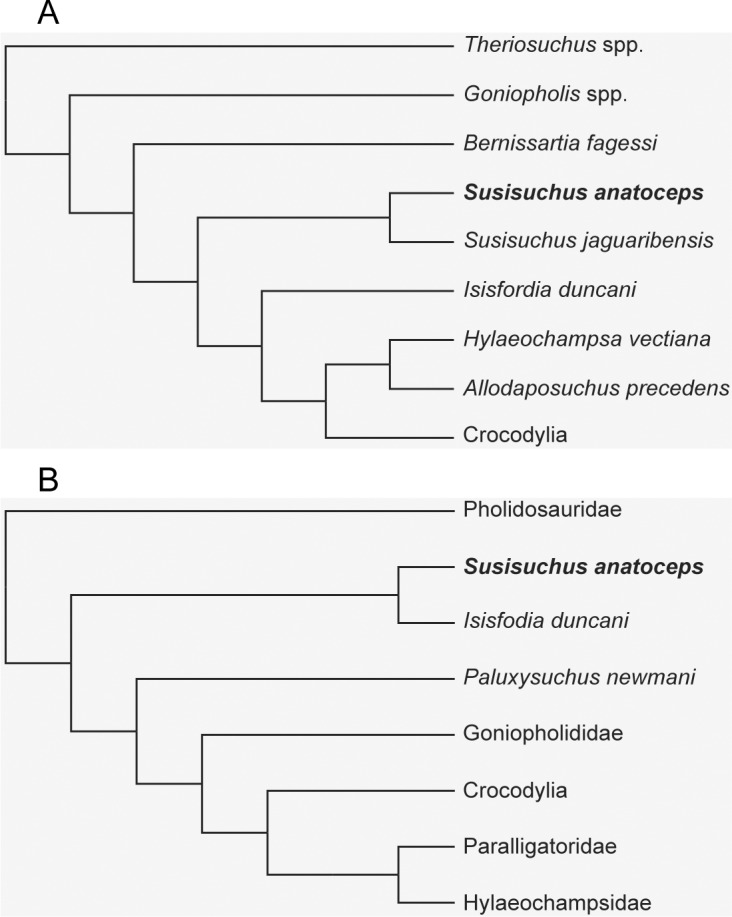
Simplified cladistic hypotheses for Neosuchia and the phylogenetic positioning of *Susisuchus anatoceps*. (A) Hypothesis of Fortier & Schultz (2009) shows susisuchids as an "advanced neosuchian" and the sister-taxon of Eusuchia. (B) Hypothesis of Turner & Pritchard (2015) displays a more basal positioning for *S*. *anatoceps* along with the Australian species *I*. *duncani*.

Among the few taxa that are represented by better preserved material is *Susisuchus anatoceps*, a small “advanced neosuchian” (Fig 1) known by well-preserved specimens from the Crato Formation (Aptian-Albian) *Konservat-Lagerstätte* of the Araripe Basin, Brazil [16–19]. A second, but less complete species of this genus, *Susisuchus jaguaribensis*, was later described from the Early Cretaceous (Berriasian-Barremian) Lima Campos Basin [20].

*S*. *anatoceps* shows a combination of plesiomorphic and derived features that makes it a key taxon for the understanding of the early evolution of the Eusuchia [16, 19, 21–23]. Some morphological innovations of the Eusuchia are already present in *S*. *antoceps*, such as the incipient procoelous cervical vertebrae [19]. Yet, other features suggest a more basal position for this taxon, excluding it from the Eusuchia. This is the case of the anterior projection of the frontal bone, which separates the nasals, the absence of both the antorbital and mandibular fenestrae, and the leveling between the quadrate condyles and the occipital condyle [16, 22]. The exquisite three-dimensional preservation of the specimens is typical from the lacustrine paleoenvironment of the Crato Formation, and favors the preservation of much of the internal bone microstructures [24].

The study of bone microstructure is a powerful tool that complements the traditional morphological descriptions and allows the inference of important information about the biology of extinct animals, such as growth rates, lifestyle adaptations, and ontogenetic stages [24–29]. The paleohistology of neosuchian taxa, however, remains still largely unexplored. Up to date, the only published thin sections regarding these animals are those of the dyrosaurids *Dyrosaurus phosphaticus* and *Guarinisuchus munizi* [28, 29]. Here we provide an histological study of the ulna and one rib of a referred specimen (MPSC R1136) of *Susisuchus anatoceps*. This is the first histological study of an “advanced neosuchian”.

## Geological Setting

The Araripe Basin is located in northeastern Brazil, in the central part of the Borborema Province [30]. It is an intracratonic basin and the most extensive of the interior basins in northeastern Brazil [31]. The stratigraphy of the Araripe Basin is very complex and remains controversial (e.g., see [30–42]). In this paper, we follow the terminology proposed by Neumann and Cabrera (1999) [38]. These authors carried out a detailed stratigraphic review of the Araripe Basin, elevating the former Santana Formation to the status of Group, and the Crato, Ipubi and Romualdo members to the status of formations (see [42] for more details). The Crato Formation is the lower most stratigraphic unit in the Santana Group [39]. It consists mainly of micritic laminated gray and cream limestones with halite pseudomorphs [43]. The Crato Formation (lacustrine-carbonatic) together with the upper part of the underlying Barbalha Formation (deltaic) constitute the lacustrine Aptian_Albian sequence of the post-rift phase of the Araripe Basin [39, 44]. The fossiliferous record of this formation is abundant and diverse [34]. The fossils are found in laminated limestones of lacustrine environments that developed under tropical, arid and semi-arid climatic conditions, with long intervals of dry weather and periodic precipitation [43]. The Crato Formation has produced an immense variety of fossils of both, fauna and flora, including plants [43, 45–48], insects [49], ostracods [50], conchostracans [51] fishes (e.g. [52, 53]), amphibians (e.g. [54, 55]), pterosaurs [56–62], crocodylomorphs [17, 19] and feathers (e.g. [63]). The preservation of is the material is often exceptional, conferring to the Crato Formation the status of *Konservat Lagerstätte* [57, 64–68].

## Materials and Methods

### Specimen

No permits were required for the present study, which complied with all regulations. The specimen MPSC R1136 is housed in the paleontological collection of the Museu de Paleontologia da Universidade Regional do Cariri (Santana do Cariri, Ceará State, Brazil). The material was previously described and assigned to the species *Susisuchus anatoceps* by Figueiredo et al. 2011 [20]. MPSC R1136 is recognized as the third specimen of *S*. *anatoceps* on the basis of at least three diagnostic features shared with the holotype, SMNK PAL 3804 (Staaliches Museum für Naturkunde Kalsruhe, Germany) [20]. Furthermore, the referred material comes from the same stratigraphic unit as the holotype, which is an important aspect for systematic purposes regarding fossils (e.g. [69, 70]). Despite the preservation of an almost complete articulated skeleton, only the middle shaft of the right ulna and one right thoracic rib were used in this study.

### Histological descriptions

We followed the osteohistological terminology of Francillon-Vieillot et al. (1990) [71] and used the phylogenetic relationships of Turner & Pritchard (2015) [23]. General features of the cross-section are described, then microstructures are discussed in detail, from the endosteal margin to the periosteal surface.

### Slide Preparation

**Fig 2:**
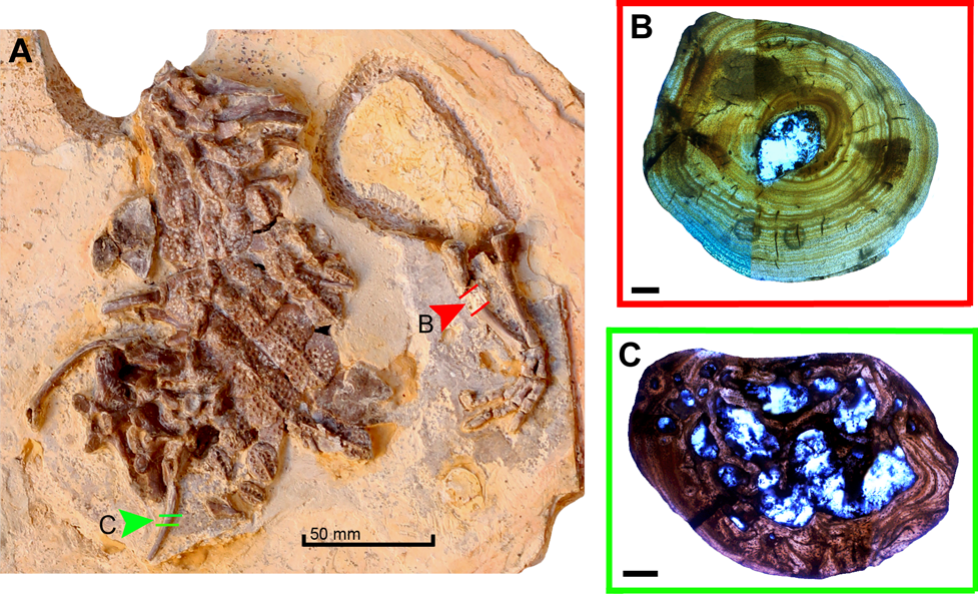
Sampled bones of MPSC R1136 with respective thin sections. (A) General view of the specimen. Red (marked B) and green (marked C) arrows (corresponding to rib and ulna respectively) indicate where the cut were made for the sample collection. (B) View of the cross section of the ulna. (C) View of the cross section of the rib. Scale bar 50 mm in A; 5 mm in B; C.

For this analysis, the mid- diaphysis of the right ulna and one thoracic rib were sectioned (Fig 2). A 0.5 cm sample was obtained from each specimen in order to prepare the histological slides.Prior to sampling, all bones were mechanically prepared with the use of airscribes and manual tools. Molds in silicon rubber (RTV CAL/N—ULTRALUB QUÍMICA LTDA, São Paulo, Brazil) and resin casts (RESAPOL T-208 catalyzed with BUTANOX M50—IBEX QUÍMICOS E COMPOSITOS, Recife, Brazil) were produced to preserve the external morphological information of the specimens. The bones were subsequently measured and photographed according to the protocol proposed by Lamm 2013 [72].

Thin sections were produced using standard fossil histology techniques [72, 73]. The samples were embedded in epoxy clear resin RESAPOL T-208, catalyzed with BUTANOX M50, and cut with a diamond-tipped blade on a saw (multiple brands). The mounting-side of the sections were wet-ground using a metallographic polishing machine (AROPOL-E, Arotec LTDA) with Arotec abrasive papers of increasing grit size (60/P60, 120/P120, 320/P400, 1200/P2500) until a final thickness of 30–60 microns was reached.

### Imaging and Image Analysis

Histological structures were observed with an optical microscope in transmitted light mode. Parallel/crossed nicols and fluorescence filters were used to enhance birefringence. Histological images were taken using an AxioCam digital sight camera (Zeiss Inc., Barcelona, Spain) mounted to an Axio Imager.M2 transmitted light microscope (Zeiss Inc. Barcelona, Spain). Images were taken at 56 and 106 total magnification.

To access Figshare online data: http://dx.doi.org/10.6084/m9.figshare.1507480

## Results

### Ulna

**Fig 3:**
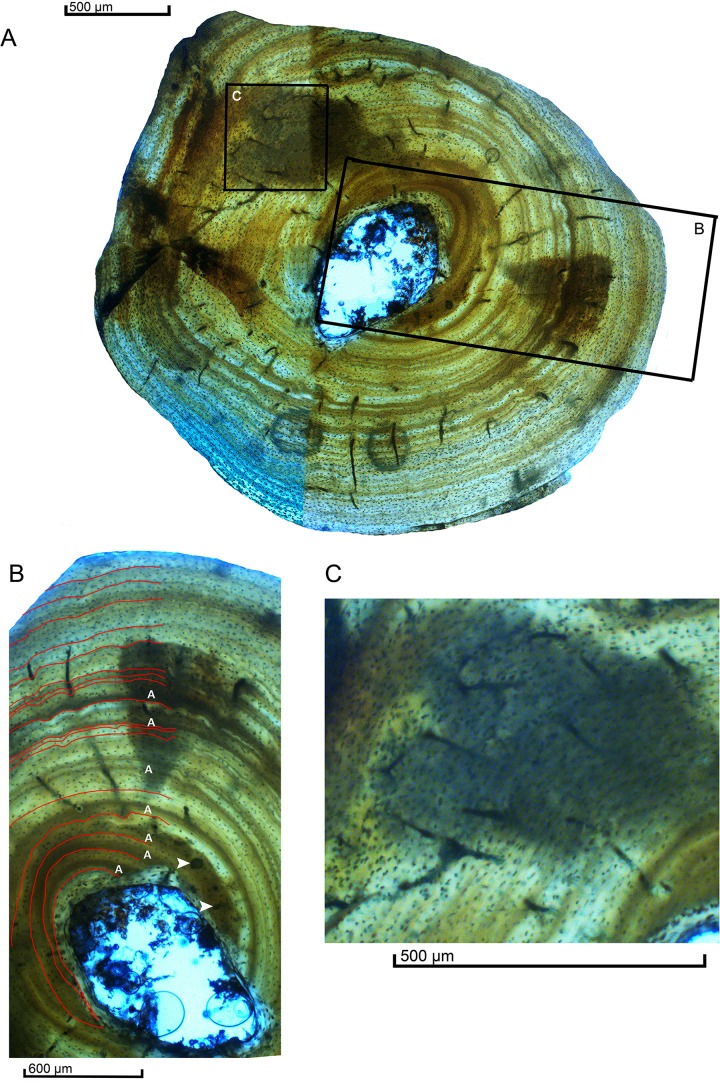
Histological characteristics of the Ulna. (A) View of the cross section. Black boxes indicate where the related images were taken. (B) View of the cortex exhibiting vascular canals parallel-fibered bone embedded with osteocyte lacunae. Seventeen simple lines of arrested growth—LAGs (partially covered by lines) can be observed from the inner cortex (endosteal region) to the outer surface (periosteal region) and seven annulus (marked by A), the white arrows indicate erosion rooms. (C) Detail of the primary bone tissue, showing the few scattered simple and anastomosed vascular canals composesing the vascular network.

The endosteal margin in the ulna is surrounded by the endosteal lamellae. The most striking feature is the complete absence of spongy tissue, giving rise to a free medullary cavity which is 730 μm in diameter (Fig 3). The marrow cavity extends to the first quadrant (superolateral portion) of the cross-section. The compact cortex is composed of primary parallel-fibred bone tissue that is 1.110 μm in diameter.The vascular network is present only in the inner and mid-portion, whereas the outer portion is free of vascularization. The vascular canals show a random distribution along the cortex, and some of these canals anastomose and run obliquely (Fig 3C). This tissue is characterized by growth cycles (zones-annuli-LAGs). A single primary osteon appears in the deep cortex between the fourth and fifth LAG. There is no evidence of secondary osteons or spongy bone. There are some small erosion rooms (Fig 3B) near the medullary cavity, which represent signal of remodeling process. These structures are more numerous in the outer cortex than in the deep cortex. The shape of the osteocytes varies along the tissue, being more flatten periostealy in comparison to the more rounded ones endostealy. Their orientation follows the same pattern of the fibrillar and lamellar organization.

The growth marks are widespread in the primary cortex. There are 5 complete growth cycles in the inner cortex (zone-annuli and LAG), followed by a thicker zonecontaining two closely annuli but no LAGs. The next growth marks is a row of three closely-spaced LAGs with zones between them. After these triple LAGs, an annulus follows it representing a low bone deposition with another growth cessation marked by a LAG. The next growth cycle starts with an annulus and is followed, again, by three close-spaced LAGs. The six last cycles are represented by zone and LAGs. There was no external fundamental system (EFS) preserved as already observed in other basal Neosuchia (see [28] for a review).

### Thoracic rib

The rib exhibits a parallel-fibered histological pattern that is similar to that of the ulna, yet it shows some important differences. In general, the cortex in the rib isthinner than in the ulna, which is 310 μm in diameter. The most notable difference is the deposition of a dense spongy tissue, which is absent in the ulna. The rib also exhibits a different pattern of cortical LAGs compared to the ulna; only five of them can be observed in the rib cortex. LAGs can be observed spreading all over the cortex.

**Fig 4:**
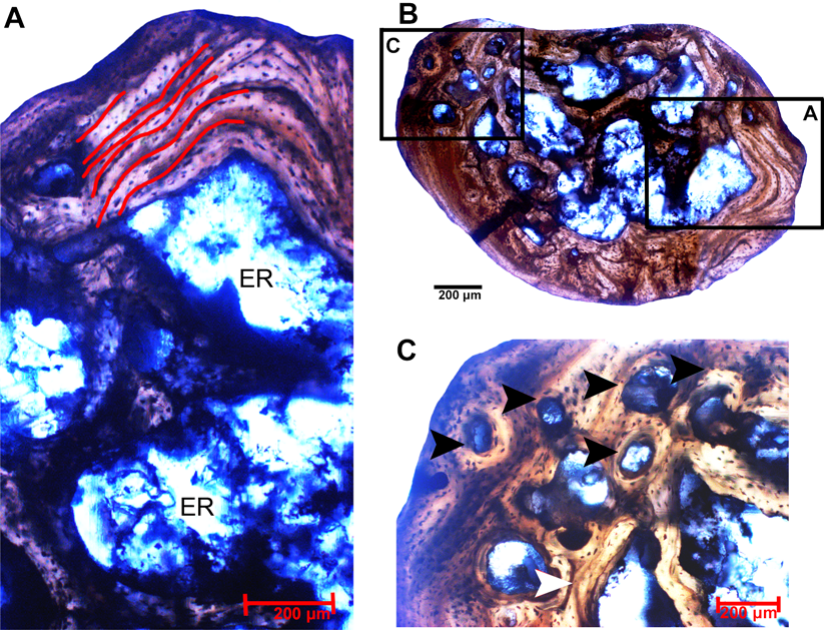
Histological characteristics of the Rib. (A) View of the cortex exhibiting parallel-fibred bone embedded with osteocyte lacunae and a deposition of spongy tissue highlighted by erosion rooms (ER). Five simple lines of arrested growth—LAGs (marked by lines) can be observed in the cortex. (B) View of the complete transect. Black boxes indicates respectively, were the related images were taken. (C) Detail of the outer cortex showing the intense remodeling process and the presence of few secondary osteons (black arrows) and an isolated simple vascular canal (red arrow).

Few secondary osteons are present. They are located in the inner and outer cortex, indicating that the process of bone remodeling was active in this individual. Therefore, the possibility of loss of other LAGs by bone resorption cannot be ignored. There was no deposition of the avascular bone lamellae called external fundamental system (Fig 4).

## Discussion

The use of paleohistology allows the identification and characterization of four general signs of biological properties in microscopic structures; i.e. ontogeny, phylogeny, biomechanics and habitat (e.g. [74]). These are influenced by age, rate of growth, physiology and other factors at different moments of the life of a given taxon, and inferences about them can be made if they are linked to comparative information [75]. Despite the diversity of crocodylomorph taxa, which show a great variety of habitats and life styles, there is a lack of knowledge about their histological patterns throughout the fossil record. Until now, the paleohistological studies of Crocodylomorpha and close-related groups range from the basal Pseudosuchia and Phytosauria [76] through Notosuchia (*Simosuchus clarki*, [77]. The Thalattosuchia [25] and Dyrosauridae [28, 29] represent the only two groups of basal Neosuchia with well-known paleohistological data. Bones of extant crocodylians have also been sectioned, including those of *Crocodylus niloticus* [78, 79], *Crocodylus johnstoni* [80], *Alligator mississipiensis* [81, 82] and *Gavialis gangeticus* [83].

### Bone Tissues and Growth Strategies

The histological pattern observed in *Susisuchus anatoceps* is parallel-fibered (PFB). This tissue is often poorly vascularized or avascular, with simple canals or primary osteons distributed randomly when present. [73,84]. In *S*. *anatoceps* the PFP cortex is avascular in the periosteal region, and the vascular canals increase endostealy towards the inner cortex with some anastomoses, as observed in both the ulna and rib. The tissue organization and their fibers can reveal rates of growth and bone depositionin organisms (e.g [85, 86]). The growth rate increases with the amount of vascularization and decreases with the degree of collagenous fibers organization, starting from fibrous to lamellar [87]. This type of tissue is deposited slower than the fibro-lamellar [85, 86]. The latter is often found in mammals, birds, synapsids, dinosaurs and pterosaurs [24, 27, 84, 88, 89].

The presence of parallel- fibered bone tissue indicates slow bone deposition. However, several moments of pause in growth are still present and evidenced by the lines of arrested growth (LAGs). LAGs are regularly formed throughout the animal’s life [90]. The annual cyclicity of LAGs have been proposed before for captive crocodylians exposed to constant temperature, diet, and photoperiod, yet they still exhibit the periodic and cyclical skeletal growth banding of their wild counterparts [91]. As far as we know from extant crocodylians, the LAGs appear to be formed in annual periodicity. In a captive-bred four-years-old *Crocodylus siamensis* three LAGs were found and one was in process of forming before its death [92]. *Crocodylus niloticus* have been shown to have LAGs formed cyclically, after the use of fluorescent markers in dermal scutes [78], as well as in a population of *Crocodylus johstoni* [80]. An exception is observed in *Alligator mississipiensis*, in which a less distinctive extra growth mark can be formed under artificially induced periods of cold or heat stress [93].

Despite the huge difference in the growth history patterns between the ulna and the rib of MPSC R1136, the latter reveals advanced remodeling process due to an enlarged medullary cavity, large erosion rooms and a thin cortex. Therefore, it could not be considered for skeletochronology. A large number of LAGs (17) and annuli (7) were found in the ulna, representing the ciclicity of the growth. The first five cycles (zone-annuli and LAG) are followed by a thicker zone with two closely annuli and no LAG in it, representing the deacrease of bone deposition. The next GM is a row of three close-spaced LAGs with zones between them. After these triple LAGs, there is an annulus, representinga period of low bone deposition rate, that ultimately terminated in a LAG. The next growth cycle starts with slow bone deposition (annulus) and is followed again by three close-spaced LAGs. The last six cycles are represented by zone and LAGs. Considering that such retention in growth occurs annually, than it is possible to infer 17 years to this individual at the moment of death. In this bone the remodeling process was just beginning with three LAGs recovered from the resorption area for the maintenance of the medullary cavity. This is consistent with the current knowledge for this group, in which LAGs formation occurs annually [78, 81, 82].

**Fig 5:**
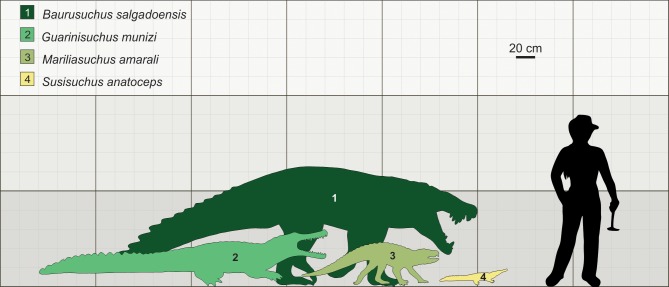
Comparative size of *Susisuchus anatoceps* to other Brazilian Cretaceous Crocodylomorphs. Comparative size diagram of Brazilian fossil crocodylomorphs, showing the dwarfism of *Susisuchus anatoceps* with 70 cm of maximum length. Ilustration by Aline M. Ghilard.

Despite the small size of the individuals assigned to *Susisuchus anatoceps*, the parallel-fibered bone with the high number of growth cycles, and the degree of remodeling of the rib, are indicative that MPSC R1136 was an animal of advanced ontogenetic stage (Fig 5). The living genera *Paleosuchus* and *Osteolaemus* are considered dwarf crocodylians, with adult average sizes ranging between 1.0 and 1.5 meters and, therefore, similar to *Susisuchus* [94]. *Paleosuchus* males reach sexual maturity when they have grown to at least 1.4 meters and females about 1.3 meters; this size category likely corresponds to 10–20 years of age [94–96].The holotype of *S*. *anatoceps* is about 60 cm in length, and was supposed to represent a young animal on the basis of some morphological features [17]. However, *S*. *anatoceps* does not reach the length observed in the extant species cited above. Based on the number of LAGs, the estimated age for MPSC R1136 is 17 years, within the age range of sexual maturity of dwarf crocodylians. Analysis of growth rates in various groups of living vertebrates suggests that, in general, small species grow more slowly than large species [97]. However, smaller species may reach their mature size earlier than larger ones [98].

Within Crocodylomorpha, some extant (*Paleosuchus* and *Osteolaemus*) and fossil (e.g. Atoposauridae and *Susisuchus*) taxa are considered dwarves when adult individuals are less than 2 m long and apparently retain a certain number of juvenile characters in adulthood, usually through pedomorphogenic processes [99,100]. This is one possible interpretation for the set of characteristics observed in *Susisuchus anatoceps*. In the original description of MPSC R1136, Figueiredo et al. (2011) [20] stated that some appendicular indicate it is a juvenile, whereas other traits observed in the osteoderms and axial skeleton were more suggestive of an adult morphology. Because these conditions are also found in the holotype of *Susisuchus*, Figueiredo et al. (2011) [20] concluded that both MPSC R1136 and the holotype were not completely mature individuals [17, 20]. Salisbury et al. (2003) [17] identify some features in the skull (e.g. large orbits, short posterior process of the quadrates, feeble ornamentation) and postcranium (e.g. poorly-torsioned humerus, indistinct muscle scars on the forelimbs) of the holotype of *S*. *anatoceps* that are associated with juveniles in most extant crocodylians, but also with mature individuals of dwarf taxa such as *Osteolaemus tetraspis* and *Paleosuchus* spp. The osteohistological features observed here agree with this last interpretation, and MPSC R1136 cannot be regarded as a young individual as previously suggested. The bone microstructure of *S*. *anatoceps* presents a pattern consistent with a late subadult animal due to the absence of EFS, and morphologically their bones show patterns of juvenile/adult transition. This suggests that *S*. *anatoceps* reaches some degree of cranial skeletal maturity before the growth of the appendicular skeleton was completed.

The absence of deposition of an external fundamental system (EFS) layer in MPSC R1136 could complicate the ontogenetic interpretation. The presence of such layers in the bones of crocodylomorpha is controversial. The EFS layer has been reported in many different taxa, such as in Lepidosauria, non-crocodylomorph pseudosuchians, Pterosauria, and Dinosauria ([81] TB Kellner 2013). However, the presence of EFS layers in crocodylomorphs is comparatively rare in the evolutionary history of the group. So far, this record of asymptotic growth has only been found in basal Pseudosuchia [76], the eusuchian *Alligator* [81] and in the neosuchian Dyrosauridae [28]. The absence of an EFS indicates that this animal had not reached full size or the end of its active growth phase at the moment of its death. Because the outermost zones are all approximately the same width and do not decrease approaching the periosteum (Fig 3B), it was likely capable of further growth potentially lasting many more years.

### Lifestyle

*S*. *anatoceps* was considered a freshwater semi-aquatic animal based on its external morphology and general *bauplan* [18, 21]. The histological pattern of a given species also provides information about the body adaptations to different life styles [25, 28, 87]. The thickening of the cortex is often considered an adaptation for buoyancy in aquatic animals [101–105]. Dense bones have been reported in aquatic animals such as the basal diapsid *Claudiosaurus*, the placodont *Placodus*, and some derived mosasaurids [87,106]. It is notable that the increase in bone mass and density are common skeletal modifications in terrestrial vertebrates transitioning to a semiaquatic existence [107]. Recently, a semiaquatic habit for the theropod dinosaur *Spinosaurus* was proposed by Ibrahim et al. (2014) [108] based on its enlarged midline display structures, the lack of free/open medullary cavities in the long bones, and increased bone density. In the extant crocodylians *Alligator mississippiensis* and *Osteolaemus tetraspis*, the heavy limbs are used to stabilize the body in water [109, 110].

The ulna of *S*. *anatoceps* has a very thick cortex and a narrow free medullary cavity, which characterizes an osteosclerotic bone pattern. This type of bone comprises an inner compaction of the bone structure [25, 111], resulting in increased skeletal mass. It is considered to play the functional role of ballast for buoyancy control and hydrostatic regulation of body trim [28, 106, 111]. If other limb bones present the same pattern observed in the ulna, the presence of an osteosclerotic limb allied to a lighter axial skeleton (represented/sampled by the rib of MPSC R1136) could be related to buoyancy control and swimming capabilities. Those features are already known for this group and this pattern is present in groups that have to maintain heavy limbs to control the position of the head above the water, which is also observed in living species [78, 81].

**Fig 6:**
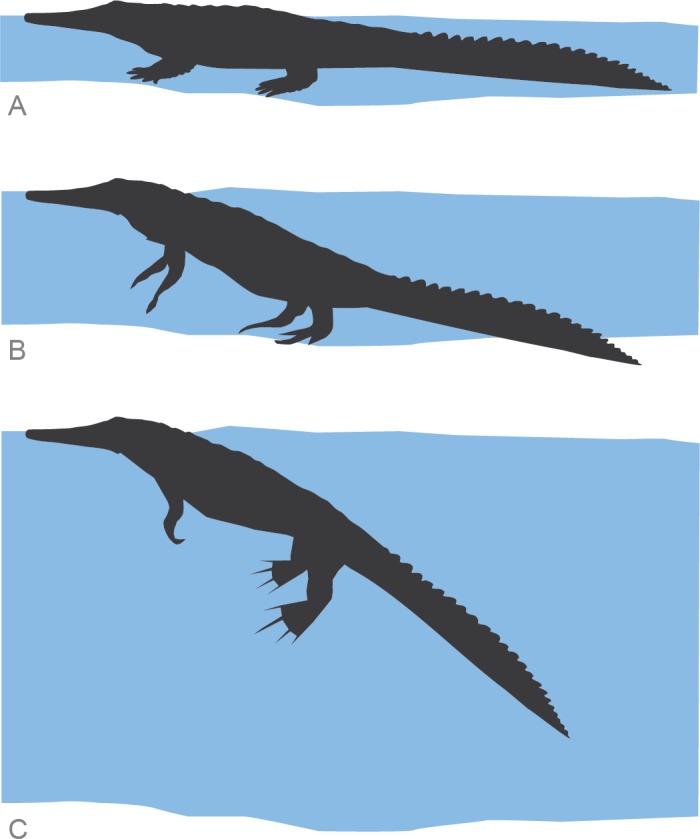
Schematic representation of resting postures of *Susisuchus anatoceps* based in extant crocodylomorphs. (A) Resting in shallow water with both body and tail contacting the bottom. (B) In shallow water when it is not able to touch the ground the hind limbs and half of the tail helping to support the animal. (C) When resting in deep water the limbs are held out nearly horizontally from the body, with fore and hind limbs extended to controls its position in buoyancy.

Crocodylians are only semiaquatic, so it may be expected that swimming in this group would be relatively expensive compared to fully aquatic animals. Distribution of body mass bone tissue is strictly related to buoyancy control, characterizing the strategies of locomotion in vertebrates [28, 78, 81]. In the extant crocodile *Crocodylus porosus*, aquatic propulsion by paddling with limbs is energetically expensive and ineffective relative to axial propulsion by tail undulation [112–115]. It seems likely that the use of the appendages, which is observed in hatchling crocodiles and in medium-sized crocodiles at low speed only, is employed to stabilize the body in the water, particularly at low speeds, rather than to contribute substantially to propulsion [116]. When *Osteolaemus tetraspis*, a species morphologically similar to *S*. *anatoceps*, is not able to touch the ground its body floats at a steep angle relative to the water surface, with the head remaining in a horizontal position [110]. The limbs are held out nearly horizontally from the body with the fore and hindlimbs extended (Fig 6). If the buoyancy is disturbed, the animal controls its position in the water by small rowing movements of the limbs [117]. This kind of resting posture has been already observed in *A*. *mississipiensis*, *C*. *niloticus*, *C*. *johnstoni*, *C*. *porosus*, *Caiman crocodilus and Gavialis gangenticus* [110]. This strategy is broadly present in recent taxa and its compatible with the distribution of the bone pattern of *S*. *anatoceps*. These observations lead to an interpretation that *Susisuchus*, like modern crocodiles, controlled buoyancy and aquatic movements by using their limbs. Despite the morphological and histological similarities between *Susisuchus* and recent taxa, more sampling on other bones of this species are needed to validate this hypothesis.

## Conclusions

Based on the parallel-fibered bone, vascular network, the high number of annual growth, the maintenance of the cortical bone in the ulna, and a highly remodeled rib with a thin cortex, *Susisuchus anatoceps* (MPSC R1136) is considered to be a late subadult individual. None of the sampled bones show evidence of EFS deposition, indicating that the growth asymptote was not reached at the time of its death. Taken together, the histological and morphological evidences presented here strongly suggest that *Susisuchus anatoceps* was a dwarf crocodylomorph and that the specimens collected to date do not represent juveniles. The distribution and number of cortical growth marksalso suggests that this species had a moderate growth rate. In the rib, the cortical tissue is thinner and remodeled, indicating that the axial skeleton was less dense than than the appendicular skeleton.

The distribution of compact and spongy tissues in the ulna suggests that *Susisuchus* could control its buoyancy and aquatic movements by using its limbs Similar to extant crocodylians. The presence of osteoclerotic bone favors a more aquatic lifestyle. However, more sampling of other bones from this species is needed to test this hypothesis.
